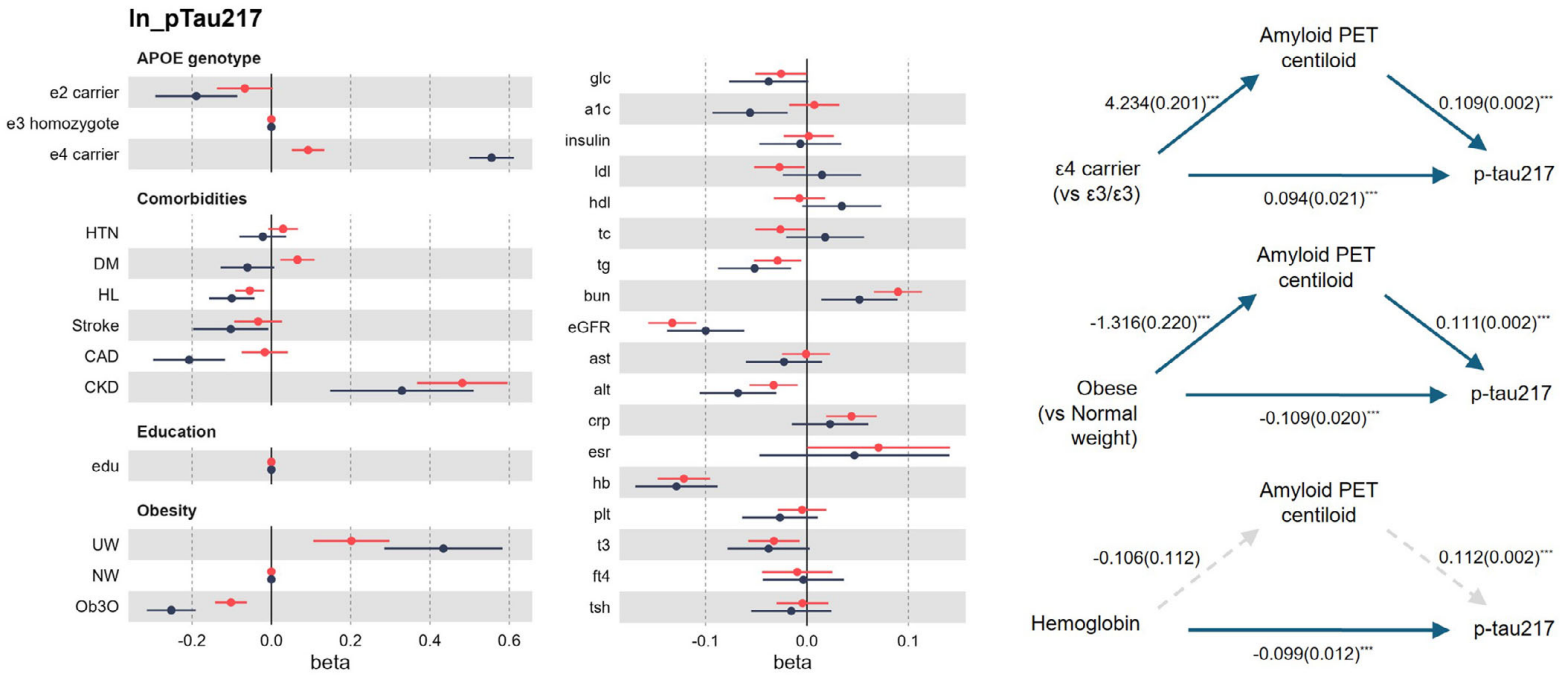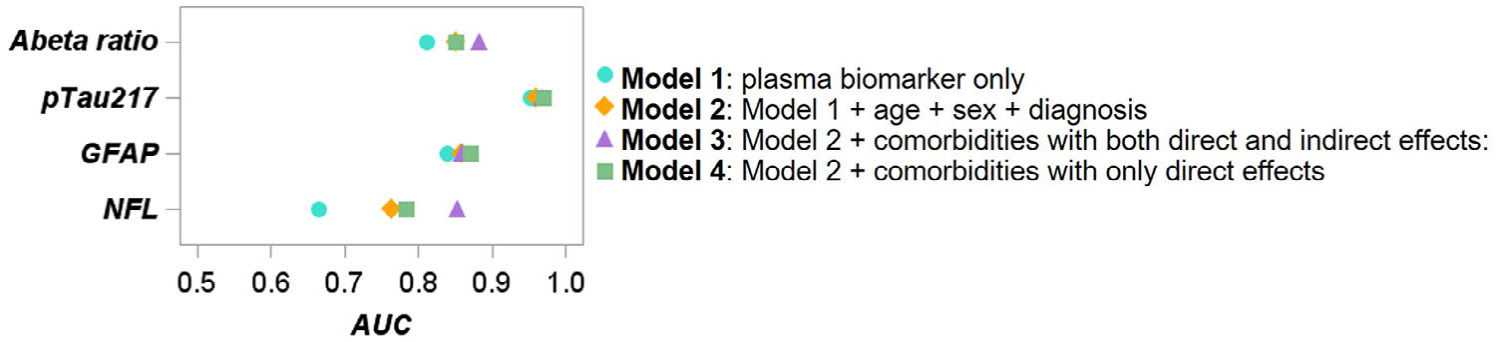# Relationships between medical comorbidities and plasma biomarkers of Alzheimer's disease reflect increased pathological markers as well as biological variabilities

**DOI:** 10.1002/alz.087856

**Published:** 2025-01-09

**Authors:** Eun Hye Lee, Sung Hoon Kang, Young Ju Kim, Henrik Zetterberg, Kaj Blennow, Fernando Gonzalez‐Ortiz, Daeun Shin, Heejin Yoo, Bo Kyoung Cheon, Jun Pyo Kim, Hee Jin Kim, Duk L. Na, Hyemin Jang, Sang Won Seo

**Affiliations:** ^1^ Samsung Medical Center, Sungkyunkwan University School of Medicine, Seoul Korea, Republic of (South); ^2^ Korea University Guro Hospital, College of Medicine Korea University, Seoul Korea, Republic of (South); ^3^ Alzheimer’s Disease Convergence Research Center, Samsung Medical Center, Seoul Korea, Republic of (South); ^4^ Department of Psychiatry and Neurochemistry, Institute of Neuroscience and Physiology, the Sahlgrenska Academy at the University of Gothenburg, Mölndal Sweden; ^5^ Department of Psychiatry and Neurochemistry, Institute of Neuroscience and Physiology, The Sahlgrenska Academy at the University of Gothenburg, Mölndal Sweden; ^6^ Clinical Neurochemistry Laboratory Sahlgrenska University Hospital, Mölndal Sweden; ^7^ University of Gothenburg, Mölndal Sweden; ^8^ SAIHST, Sungkyunkwan University, Seoul Korea, Republic of (South); ^9^ Neuroscience Center, Samsung Medical Center, Seoul Korea, Republic of (South); ^10^ Sungkyunkwan University, Suwon Korea, Republic of (South)

## Abstract

**Background:**

Plasma biomarkers for Alzheimer’s disease (AD) have demonstrated their accuracy as diagnostic tools, suggesting their impending integration into clinical practice. Medical comorbidities might not only affect AD pathological burdens but also cause variability of plasma biomarkers by affecting their transfer via blood brain barriers. In the present study, we aimed to determine which comorbidities might affect plasma biomarkers with (real effects) or without (biological variability) AD pathological burdens measured by β‐amyloid (Aβ) uptakes on PET.

**Method:**

We recruited 2,935 participants including cognitively unimpaired (n=673), mild cognitive impairment (n=1439), dementia of Alzheimer’s type (n=611), and subcortical vascular cognitive impairment (n=190) from Korea‐Registries to Overcome and Accelerate Dementia research. All of them underwent amyloid PET imaging. Their plasma Aβ40, Aβ42, p‐tau217, GFAP and NfL levels were measured by a single molecular assay on a single platform.

**Result:**

Various medical comorbidities were predictive of decreased or increased plasma biomarker levels after controlling for Aβ uptakes on PET. By using these medical comorbidities as predictors and Aβ uptakes as mediators, we performed the mediation analyses. Aβ uptakes partially mediated the relationships between various comorbidities and plasma biomarkers: APOE ε4 for Aβ42/40 ratio, p‐tau217, GFAP and NfL; body mass index (BMI) status for p‐tau217 GFAP and NfL. Various comorbidities also directly affected plasma biomarkers levels: estimated glomerular filtration rate (eGFR) for Aβ42/40 ratio, p‐tau217, GFAP and NfL; chronic kidney disease, hemoglobin, C‐reactive protein, and T3 for p‐tau217, GFAP and NfL; erythrocyte sedimentation rate (ESR) for GFAP and NfL. Finally, adding the comorbidities with direct and indirect effects to the covariates increased the AUCs of Aβ42/40 ratio (0.851 to 0.882) and NfL levels (0.764 to 0.853), but not those of pTau217 and GFAP. However, adding the comorbidities with only direct effects to the covariates did not increase the AUCs of any plasma biomarkers.

**Conclusion:**

Our findings revealed that various medical comorbidities could influence plasma biomarker levels, both directly and through mediation of AD pathology. Therefore, it is important to consider in terms of clinical application and interpretation of plasma biomarkers results in clinical practice.